# NOTCH1 Is Aberrantly Activated in Chronic Lymphocytic Leukemia Hematopoietic Stem Cells

**DOI:** 10.3389/fonc.2018.00105

**Published:** 2018-04-20

**Authors:** Mauro Di Ianni, Stefano Baldoni, Beatrice Del Papa, Patrizia Aureli, Erica Dorillo, Filomena De Falco, Elisa Albi, Emanuela Varasano, Ambra Di Tommaso, Raffaella Giancola, Patrizia Accorsi, Gianluca Rotta, Chiara Rompietti, Estevão Carlos Silva Barcelos, Antonio Francesco Campese, Paolo Di Bartolomeo, Isabella Screpanti, Emanuela Rosati, Franca Falzetti, Paolo Sportoletti

**Affiliations:** ^1^Department of Medicine and Aging Sciences, University of Chieti Pescara, Chieti, Italy; ^2^Department of Hematology, Transfusion Medicine and Biotechnologies, Ospedale Civile, Pescara, Italy; ^3^Department of Life, Health and Environmental Sciences, Hematology Section, University of L’Aquila, L’Aquila, Italy; ^4^Institute of Hematology-Centro di Ricerche Emato-Oncologiche (CREO), University of Perugia, Perugia, Italy; ^5^BD Biosciences, San Jose, Italy; ^6^Universidade Federal do Espírito Santo, Vitória, Brazil; ^7^Department of Molecular Medicine, Sapienza, University of Rome, Rome, Italy; ^8^Department of Experimental Medicine, Biosciences and Medical Embriology Section, University of Perugia, Perugia, Italy

**Keywords:** notch signaling, chronic lymphocytic leukemia, hematopoietic stem cells, NOTCH1 mutation, CD34+ cells

## Abstract

To investigate chronic lymphocytic leukemia (CLL)-initiating cells, we assessed *NOTCH1* mutation/expression in hematopoietic stem cells (HSCs). In *NOTCH1-*mutated CLL, we detected subclonal mutations in 57% CD34+/CD38− HSCs. *NOTCH1* mutation was present in 66% CD34+/CD38+ progenitor cells displaying an increased mutational burden compared to HSCs. Flow cytometric analysis revealed significantly higher NOTCH1 activation in CD34+/CD38− and CD34+/CD38+ cells from CLL patients, regardless *NOTCH1* mutation compared to healthy donors. Activated NOTCH1 resulted in overexpression of the NOTCH1 target c-MYC. We conclude that activated NOTCH1 is an early event in CLL that may contribute to aberrant HSCs in this disease.

## Introduction

Chronic lymphocytic leukemia (CLL) is a mature B cell malignancy characterized by accumulation of clonal B cells in blood, bone marrow (BM), and lymphoid tissues. The search for CLL-initiating cells has never been successful. While several cell types have been suggested as giving rise to CLL, yet, there is no consensus as to its normal cellular counterpart (1). CLL cells have monoclonal immunoglobulin gene rearrangements, suggesting that lymphoid malignant stem cells originate after cells have committed to the lymphoid lineage. More recently, it has been reported that hematopoietic stem cells (HSCs) from CLL patients display the propensity to generate clonal B cells, suggesting the involvement of HSCs in lymphoid leukemogenesis (2).

In 2009, we first identified *NOTCH1* mutations in CLL (3) and provided data on the adverse prognostic outcome associated with mutated *NOTCH1* (4). More recently, independent studies confirmed the presence and the prognostic relevance of *NOTCH1* mutations in CLL patients (5). All mutations resulted in NOTCH1 impaired degradation that led to NOTCH1 deregulated signaling, indicating that mutations could contribute to increase NOTCH activation in CLL (6, 7). Recently, analyses of peripheral blood CD34+CD19− cells and BM hematopoietic progenitors revealed *NOTCH1* mutation in some CLL samples (8, 9). Conversely, others failed to demonstrate the presence of *NOTCH1* mutations in circulating CD34+ cells from *NOTCH1* mutated patients (10). In lymph node, CLL cells show NOTCH1 activation independent of mutation (11) and recent evidence have shown non-mutational NOTCH1 signaling with anti-apoptotic effects in peripheral blood CLL cells (12). However, the role of NOTCH1 signaling in the HSC compartment of CLL is still unknown.

## Materials and Methods

### Cell Separation and Flow Cytometry

We collected 28 BM samples including 21 CLL patients (15 NOTCH1 mutated and 6 NOTCH1 wild type) and 7 healthy donors (HDs). BM and peripheral blood (PB) cells were collected under signed informed consent in accordance with Declaration of Helsinki and the Institutional Review Board of University of Perugia. Patient’s characteristics are described in Table S1 in Supplementary Material. BM cells from 10 *NOTCH1* mutated CLL were single and/or double-sorted into CD34+CD38− HSCs and CD34+CD38+ progenitor fraction containing myeloid and lymphoid progenitors. Briefly, BM mononuclear cells were separated by Ficoll–Hypaque density gradient centrifugation. Flow cytometric analysis and cell-sorting were performed using the following antibodies: PE anti-CD34 and PC5 anti-CD38 (Beckman Coulter), PerCP-Cy5.5 anti-CD34, FITC anti-CD19, PE-Cy7 anti-CD38, APC-H7 anti-CD10, V450 anti-CD3, and V500 anti-CD45 (BD Biosciences). NOTCH1 ICN on sorted populations was performed using PE anti-NOTCH1 (mN1A) (eBioscience). Cells were analyzed using a FACSCanto II and sorted using a FACS Aria III cell sorter (BD Biosciences).

### Direct Sanger Sequencing

*NOTCH1* gene mutational analysis was performed by directional sequencing of PCR fragments from genomic DNA. Primers and PCR conditions were as previously described (13).

### Allele-Specific PCR (AS-PCR), Droplet Digital PCR (ddPCR), RT-PCR

Allele-specific PCR was performed accordingly to a previously published protocol (13). ddPCR NOTCH1 probes assays (dHsaCP2500500 and dHsaCP2500501 Bio-Rad) were used to determinate the allelic burden of NOTCH1 in sorted cells. The droplet generated included DNA, Notch1 probes assays (1×), and ddPCR Supermix (2×) for Probes (no dUTP) (Bio-Rad). The mix was amplified by PCR and analyzed by QX200 Droplet Reader (Bio-Rad). Scatterplots depicting ddPCR results specifically for the NOTCH1 mutation assay. Real-time PCR analysis of c-MYC and Hes1 gene expression was performed in immunoselected CD34+ BM cells. RNA was extracted using RNeasy Plus Kits (Qiagen, Hilden, Germany), and cDNA was obtained using Prime Script RT Master Mix (Takara Bio, Dalian, China). Real-time qPCR was performed with PCR Master Mix Power SYBER Green (Applied Biosystem, Warrington, UK) using the 7900HT fast Real-Time PCR System (Applied Biosystem). The primers sequence were F:5′-CTTCTCTCCGTCCTCGGATTCT-3′ and R:5′- GAAGGTGATCCAGACTCTGACCTT-3′ for c-Myc, F:5′-AAGAAAGATAGCTCGCGGCAT-3′ and R:5′-CCAGCACACTTGGGTCTGT-3′ for Hes1 and F:5′- ATGGGGAAGGTGAAGGTCG-3′ and R:5′- GGGGTCATTGATGGCAACAATA-3′ for GAPDH. Relative fold change was normalized to GAPDH and calculated using the 2^−ΔΔCt^ method.

### Western Blot Analysis

Whole-cell lysates extracted from BM CD34+ cells (5 × 10^5^) of CLL patients and HDs were analyzed by western blot using an anti-NOTCH1 antibody (clone bTAN20) able to detect the 300-kDa inactive precursor (FL), the 120-kDa transmembrane/cytoplasmic/cytoplasmic (TM) subunit, and the active 100-kDa intracellular domain. Whole-cell lysates (3 µg) isolated from peripheral blood CD5+CD19+ CLL cells of *NOTCH1* mutated patients were used as positive control.

### Statistical Analysis

Statistical analyses were performed with GraphPad (GrapdhPad Software Inc., La Jolla, CA, USA). In the text, data are presented as mean ± SD and statistical differences between mean values were evaluated using the Student’s *t*-test and Mann–Whitney test.

## Results

### HSCs From CLL Patients Showed NOTCH1 Mutation

**Figure 1:**
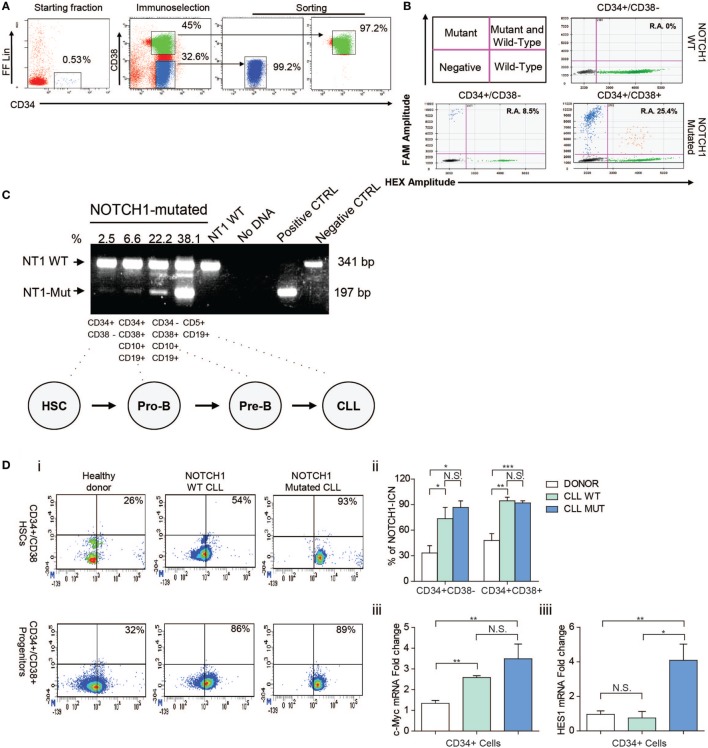
Analysis of *NOTCH1* gene mutation and signaling in bone marrow (BM) hematopoietic stem cells (HSCs) and progenitors cells. **(A)** FACS-setting used for HSCs and progenitor cells sorting purification of one representative NOTCH1-mutated patient. Prospective cell separation included immunoselection (middle plot) and sorting (right plot) to ensure purity and lack of chronic lymphocytic leukemia (CLL) cell contamination for *NOTCH1* mutation analysis. **(B)** Droplet digital PCR. Upper left panel is a schematic representation of positive and negative droplet distribution according to the fluorophore threshold indicated in magenta lines. Upper right and lower panels are representative scatterplots of wild type and NOTCH1 mutated HSCs and progenitor cells, respectively. **(C)** Results of the allele-specific PCR assay for delCT *NOTCH1* mutation in HSCs (CD34+CD38−), pro-B progenitors (CD34+CD38+CD10+CD19+), pre-B cells (CD34−CD38+CD10+CD19+), and B neoplastic CLL (CD5+CD19+) from one *NOTCH1*-mutated CLL sample. CD5+CD19+ cells from a NOTCH1-WT patient were used as negative control and showed a normal band of 341 bp. Samples bearing the delCT NOTCH1 mutation showed an additional mutant band of 197 bp. **(D)** (i) Representative dot plots of healthy control, NOTCH1 wild type, and NOTCH1 mutated CLL BM samples showing expression of NOTCH1-ICN on CD34+/CD38− HSCs and CD34+/CD38+ progenitors compartments. (ii) Bar graphs show the means ± SD of the percentage of NOTCH1-ICN positive cells. *p < 0.05, **p < 0.01, ***p < 0.001 according to Student’s *t*-test; (iii) real-time PCR analysis of c-MYC and Hes1 gene expression in CD34+ BM cells. mRNA levels were normalized to GAPDH and represented as fold change using healthy control cells as a reference.

The mean proportion of BM CD34+ cells before enrichment was 0.75 ± 0.44%. After the FACS sorting procedures, the mean purity of CD34+/CD38− cells was 94.58 ± 3.52% and CD34+/CD38+ cells were 98.12 ± 1.34% (Figure 1A).

We initially analyzed the NOTCH1 mutational hotspot by Sanger sequencing. The CD34+/CD38− fraction did not contain NOTCH1-mutated cells. Then, a high sensitivity AS-PCR assay for the *NOTCH1* mutation (13) indicated the presence of small HSCs mutated clones in 57% CLL samples. Densitometric analysis revealed a mean 6.4 ± 4.7% *NOTCH1* mutant allelic burden. Moreover, we used a ddPCR assay to validate *NOTCH1* mutational data in HSCs by a more quantitative method. We confirmed *NOTCH1* mutations in two HSCs samples for which DNA was available that display an allelic ratio of 2.6 and 8.5%, respectively (Figure 1B). Altogether, these data confirm that *NOTCH1* mutation is an early event in CLL hematopoiesis in a fraction of patients. Additionally, we measured the *NOTCH1* mutational burden along specific stages of HSC differentiation in *NOTCH1-*mutated CLL patients. The mean percentage of the mutant allele progressively increased from 6.4 ± 4.7% in CD34+CD38− to 14.9 ± 11.3% in CD34+CD38+CD10+CD19+ cells, 22.7 ± 6.5% in CD34−CD38+CD10+CD19+ cells and 40.5 ± 4.3% in neoplastic CD5+CD19+ cells (Figure 1C). The analysis of the rearrangement status of the IgH gene revealed in both NOTCH1 mutated and NOTCH1 unmutated CD34+CD38− HSCs the presence of a germline configuration in the half of the samples while the other 50% showed a clonal VDJ.

### HSCs From CLL Patients Have NOTCH1 Aberrantly Activated Also in Unmutated NOTCH1 Patients

Thus, we analyzed the NOTCH1 signaling status in HSCs and progenitor cells of NOTCH1-mutated and unmutated CLL samples. Physiologically, the active intracellular domain (ICN) of NOTCH1 accumulates in cells with activated NOTCH1 signaling as a result of a cleavage of the transmembrane (TM) subunit made by the y-secretase complex (14). Here, we used flow cytometry to quantitate the percentage of active NOTCH1-ICN in CD34+/CD38− HSCs and CD34+CD38+ progenitors from the BM of CLL patients and HDs, used as control. As shown in Figure 1Di,ii, NOTCH1-ICN was significantly higher in CLL samples regardless the *NOTCH1* mutational status compared to non-leukemic samples. Indeed, the mean percentage of CD34+/CD38−/NOTCH1-ICN+ and CD34+/CD38+/NOTCH1-ICN+ populations in NOTCH1 WT and mutated CLL was significantly higher than HDs samples (73.4 ± 22.9 and 83 ± 16.4 vs 33.3 ± 14.8%; 94.4 ± 7.3 and 92.8 ± 4.3 vs 47.9 ± 13.8%, respectively).

To demonstrate the capability of CD34+ CLL cells to activate NOTCH1 signaling pathway, we analyzed the levels of NOTCH1 downstream transcriptional target gene. It has been demonstrated that NOTCH1 controls c-MYC expression in mature CLL cells overexpressing the NOTCH1-ICN (12). Thus, using quantitative reverse transcription-PCR, we found significantly higher mRNA expression levels of c-MYC in CD34+ cells from NOTCH1 mutated and WT CLL samples compared to HD (3.5 ± 0.7 and 2.6 ± 0.08 vs 1.3 ± 0.1) (Figure 1Diii). In addition, we showed higher Hes1 expression in CD34 + cells from CLL patients compared to HD (4.2 ± 1.1 vs 1.1 ± 0.2) (Figure 1Diii), in line with upregulated NOTCH1 pathway.

**Figure 2:**
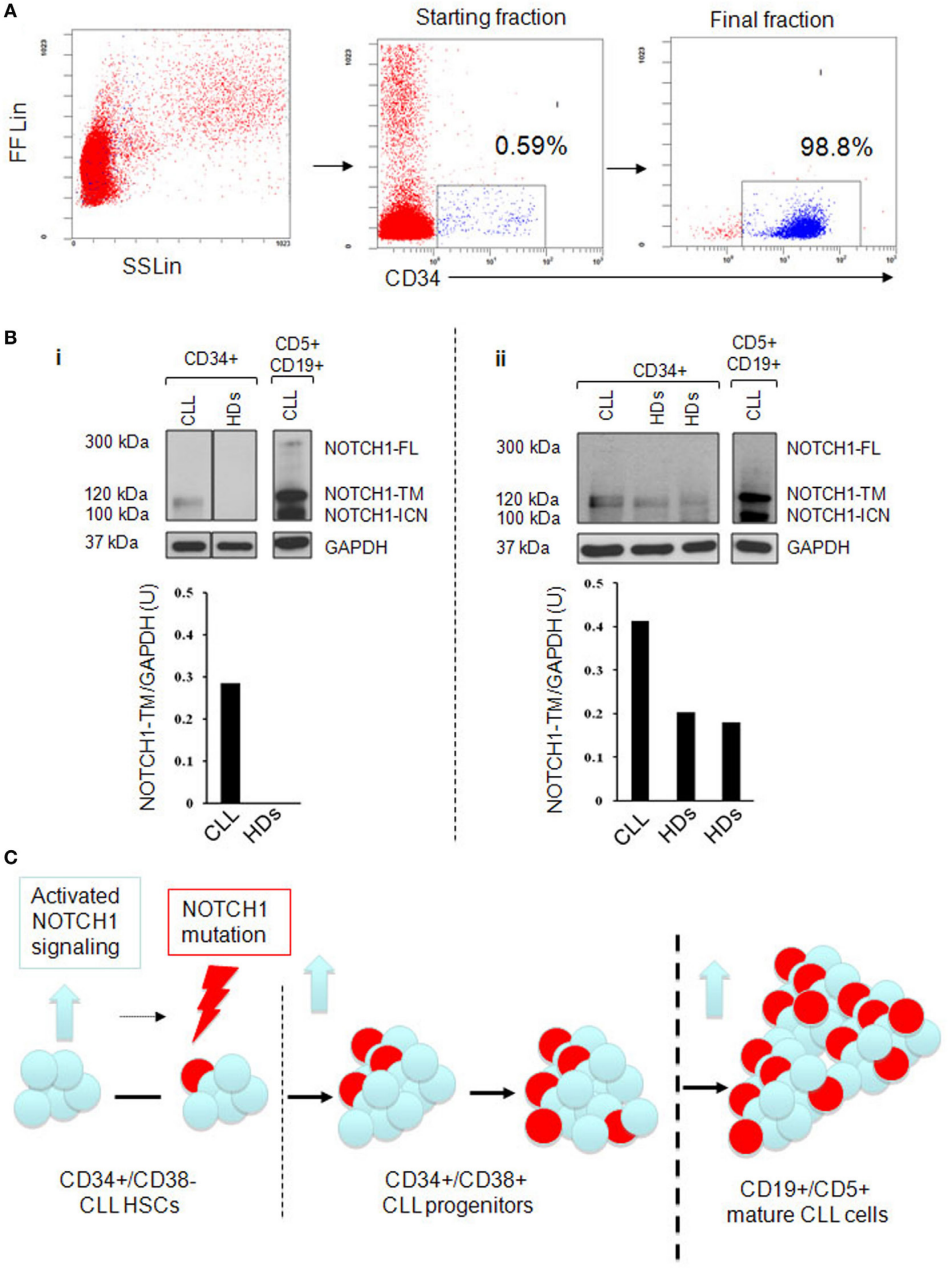
Analysis of NOTCH1-TM expression in immunoselected CD34+ bone marrow (BM) cells. **(A)** Immunomagnetic cell sorting purification and phenotypic characterization of CD34+ cells from a representative chronic lymphocytic leukemia (CLL) BM used for NOTCH1 protein expression analysis. **(B)** (i and ii) Western blot analysis. Vertical lines indicate realignment of the same blot imaging. Protein loading was assessed by reprobing the blots with an anti-GAPDH antibody. The density of the bands corresponding to NOTCH1-TM was evaluated by densitometric analysis. Densitometry units (U) were calculated relative to GAPDH. Results revealed that CD34+ samples from CLL patients always expressed the NOTCH1-TM protein. **(C)** Schematic representation of clonal evolution during the development of CLL starting from hematopoietic stem cells (HSCs). NOTCH1 is found to be active at early stage of hematopoiesis (blue arrow) together with NOTCH1 mutations (lightning arrow) to which activated signaling might contribute. NOTCH1 activation persists and the mutated clone expands as the cell commit to mature CD19+CD5+CLL.

Next, we analyzed whether the higher levels of NOTCH1 activation in HSCs and progenitors CLL samples were accompanied with increased NOTCH1 expression. We analyzed by western blot the expression levels of NOTCH1-TM subunit in CD34+ cells from BM aspirates of four NOTCH1-mutated CLL patients and three HDs (6). The median purity of immunoselected CD34+ cells was 97.3% (range 74–99.5%; Figure 2A). Results revealed that CD34+ samples from CLL patients always expressed the NOTCH1-TM protein. Conversely, in CD34+ cells from HDs, NOTCH1-TM was either absent or expressed at lower levels than those observed in CLL samples (Figure 2Bi,ii). These data demonstrated that high levels of NOTCH1 signaling activation correlated with NOTCH1-TM overexpression in the CD34+ hematopoietic compartment of CLL.

## Discussion

The Notch pathway is genetically altered in a large number of hematopoietic and solid tumors (15). We recently reported that activating mutations of *NOTCH1* are recurrently associated with CLL and predict poor outcome (3, 4). The identification of a somatic *NOTCH1* mutation could help providing additional information on the cellular origin of CLL. Sanger sequencing analysis (13) failed to detect mutations in CD34+/CD38− HSCs fractions of CLL patients. However, the sensitivity of this method does not allow the identification of a mutation whose allelic representation is <10%. Thus, we used more sensitive PCR based methods in order to verify results obtained with direct sequencing.

The analysis of CD34+/CD38+ progenitors detected the *NOTCH1* mutation in 57% of the samples. Damm et al. (8) described a similar frequency of *NOTCH1* mutation in multipotent progenitors of CLL samples performing NGS analysis on rare CD34+CD19− peripheral blood cells. Recently, the same analysis was conducted in immunoselected CD34+ BM cells that resulted *NOTCH1*-mutated in 8 out of 13 CLL samples (9). Here, we used two high sensitive PCR assays specific for *NOTCH1* mutation and employed FACS sorted CD34+CD38+ cells from BM aspirates. Additionally, we showed here that the *NOTCH1* mutational burden increased along specific stages of HSC differentiation in *NOTCH1-*mutated CLL patients. This suggests that the *NOTCH1* lesion is selected and expands during HSC differentiation toward a B neoplastic cell, thus strengthening the hypothesis that the genetic alteration is an initial event associated with the stepwise malignant transformation of CLL.

In normal human BM, CD34+CD38− populations do not rearrange the IgH gene (16), thus raising issues on the molecular purity of the sorted hematopoietic cell fractions presenting a VDJ rearrangement. Nevertheless, the high purity of HSC double-sorted samples together with the sensitivity of the IgH method (5%) limited the risk of detecting small fractions of contaminating neoplastic B cells. The alternative option is that IgH rearrangement is the expression of a B neoplastic transactivation at the HSC level.

Recently, common nonmutational NOTCH1 activation has been described in mature CLL cells (12) raising the question of whether the same condition is present in HSCs to determine their aberrant behavior. The Notch1 signaling has been extensively analyzed in the contest of embryonic hematopoiesis. *Ex vivo* approaches suggest that Notch signaling can expand HSCs, raising the question of whether this is a physiologic Notch function. Gerhardt et al. (17) correlated NOTCH1 with hematopoiesis in animal models and identified cell-autonomous functions for Notch1 signaling in fetal HSCs homeostasis. The present study indicated that the pool of CD34+ cells, including HSC and progenitor compartments, tend to have NOTCH1 aberrantly expressed and activated in CLL patients compared to HDs. NOTCH1 deregulation and overepression of c-Myc are independent of NOTCH1 mutational status. These data clearly show that expansion of the leukemic stem cell clone does not necessarily require a mutation to upregulate the NOTCH1 signaling, suggesting the presence of extrinsic factors from the BM HSC niche that are capable of stimulating and promoting CLL-initiating cell clone expansion. In CLL patients, BM mesenchimal cells express different ligands, which might play a role in NOTCH1 activation (18). However, additional studies are warranted to compare the levels and type of these ligands in the BM of healthy people vs CLL patients. Alternatively, CLL-HSCs may have cell-intrinsic mechanisms activating NOTCH1, which involve alterations of *NOTCH1* pathway regulators (19, 20) or aberrant regulation of NOTCH1 receptor recycling (21). This selective pressure might contribute to the onset of specific NOTCH1 mutations in a DNA context that is prone to spontaneous microdeletion (5) (Figure 2C).

Our discovery of *NOTCH1* deregulated signal and mutations in CLL-HSC have significant therapeutic implications in this disease. A variety of approaches was used to inhibit NOTCH1 for cancer therapy, including presenilin γ-secretasi inhibtors, trafficking modulators (22), and blocking antibodies. In the next future, it will be interesting to evaluate the effects of these anti-NOTCH1 drugs in the development of CD34+CD38− and CD34+CD38+ CLL populations.

In conclusion, our data confirmed the presence of *NOTCH1* mutations in HSCs of CLL patients and showed for the first time a common nonmutational NOTCH1 activation occurring early in CLL hematopoiesis and represent a rationale for the use of therapies targeting the NOTCH1 signaling in CLL aimed to inhibit the survival of CLL-initiating cells.

## Ethics Statement

This study was carried out in accordance with the recommendations of University/Hospital of Perugia guidelines, Ethics Committee of Perugia, with written informed consent from all subjects. All subjects gave written informed consent in accordance with the Declaration of Helsinki. The protocol was approved by the Ethics Committee of Perugia.

## Author Contributions

MDI and PS designed experiments; SB, BDP, PA, ED, FDF, ADT, RG, PA, GR, CR, ESB, and AFC performed experiments and analyzed data; EA and FF contributed to samples collection; MDI, ER, BDP, IS, and PS wrote the manuscript.

## Conflict of Interest Statement

The authors declare that the research was conducted in the absence of any commercial or financial relationships that could be construed as a potential conflict of interest.

## References

[1] Chiorazzi N Ferrarini M. Cellular origin(s) of chronic lymphocytic leukemia: cautionary notes and additional considerations and possibilities. Br J Haematol (2011) 117:1781–91. 10.1182/blood-2010-07-155663 21148333 PMC3056635

[2] Kikushige Y Ishikawa F Miyamoto T Shima T Urata S Yoshimoto G Self-renewing hematopoietic stem cell is the primary target in pathogenesis of human chronic lymphocytic leukemia. Cancer Cell (2011) 20:246–59. 10.1016/j.ccr.2011.06.029 21840488

[3] Di Ianni M Baldoni S Rosati E Ciurnelli R Cavalli L Martelli MF A new genetic lesion in B-CLL: a NOTCH1 PEST domain mutation. Br J Haematol (2009) 146:689–91. 10.1111/j.1365-2141.2009.07816.x 19604236

[4] Sportoletti P Baldoni S Cavalli L Del Papa B Bonifacio E Ciurnelli R NOTCH1 PEST domain mutation is an adverse prognostic factor in B-CLL. Br J Haematol (2010) 151:404–6. 10.1111/j.1365-2141.2010.08368.x 20813007

[5] South AP Cho RJ Aster JC. The double-edged sword of Notch signaling in cancer. Sem Cell Dev Biol (2012) 23:458–64. 10.1016/j.semcdb.2012.01.017 22309843 PMC3360804

[6] Rosati E Sabatini R Rampino G Tabilio A Di Ianni M Fettucciari K Constitutively activated Notch signaling is involved in survival and apoptosis resistance of B-CLL cells. Blood (2009) 113:856–65. 10.1182/blood-2008-02-139725 18796623

[7] De Falco F Sabatini R Del Papa B Falzetti F Di Ianni M Sportoletti P Notch signaling sustains the expression of Mcl-1 and the activity of eIF4E to promote cell survival in CLL. Oncotarget (2015) 6:16559–72. 10.18632/oncotarget.4116 26041884 PMC4599289

[8] Damm F Mylonas E Cosson A Yoshida K Della Valle V Mouly E Acquired initiating mutations in early hematopoietic cells of CLL patients. Cancer Discov (2014) 4:1088–101. 10.1158/2159-8290.CD-14-0104 24920063

[9] Quijada-Álamo M Hernández-Sánchez M Robledo C Hernández-Sánchez JM Benito R Montaño A Next-generation sequencing and FISH studies reveal the appearance of gene mutations and chromosomal abnormalities in hematopoietic progenitors in chronic lymphocytic leukemia. J Hematol Oncol (2017) 10:83. 10.1186/s13045-017-0450-y 28399885 PMC5387353

[10] Rossi FM Zucchetto A Tissino E Dal Bo M Bomben R Caldana C CD49d expression identifies a chronic-lymphocytic leukemia subset with high levels of mobilized circulating CD34(+) hemopoietic progenitors cells. Leukemia (2014) 28:705–8. 10.1038/leu.2013.331 24202404

[11] Kluk MJ Ashworth T Wang H Knoechel B Mason EF Morgan EA Gauging NOTCH1 activation in cancer using immunohistochemistry. PLoS One (2013) 8:e67306. 10.1371/journal.pone.0067306 23825651 PMC3688991

[12] Fabbri G Holmes AB Viganotti M Scuoppo C Belver L Herranz D Common nonmutational NOTCH1 activation in chronic lymphocytic leukemia. Proc Natl Acad Sci U S A (2017) 114:E2911–9. 10.1073/pnas.1702564114 28314854 PMC5389283

[13] Sportoletti P Baldoni S Del Papa B Aureli P Dorillo E Ruggeri L A revised NOTCH1 mutation frequency still impacts survival while the allele burden predicts early progression in chronic lymphocytic leukemia. Leukemia (2014) 28:436–9. 10.1038/leu.2013.289 24177259

[14] De Falco F Sabatini R Falzetti F Di Ianni M Sportoletti P Baldoni S Constitutive phosphorylation of the active Notch1 intracellular domain in chronic lymphocytic leukemia cells with NOTCH1 mutation. Leukemia (2015) 29:994–8. 10.1038/leu.2014.329 25425197

[15] Ntziachristos P Lim JS Sage J Aifantis I. From fly wings to targeted cancer therapies: a centennial for notch signaling. Cancer Cell (2014) 25:318–34. 10.1016/j.ccr.2014.02.018 24651013 PMC4040351

[16] Davi F Faili A Gritti C Blanc C Laurent S Sutton L Early onset of immunoglobulin heavy chain gene rearrangments in normal human bone marrow CD34+ cells. Blood (1997) 90:4014–21. 9354670

[17] Gerhardt DM Pajcini KV D’Altri T Tu L Jain R Xu L The Notch1 transcriptional activation domain is required for development and reveals a novel role for Notch1 signaling in fetal hematopoietic stem cells. Genes Dev (2014) 28:576–93. 10.1101/gad.227496.113 24637115 PMC3967047

[18] Nwabo Kamdje AH Bassi G Pacelli L Malpeli G Amati E Nichele I Role of stromal cell-mediated notch signaling in CLL resistance to chemotherapy. Blood Cancer J (2012) 2(5):e73. 10.1038/bcj.2012.17 22829975 PMC3366070

[19] Baldoni S Sportoletti P Del Papa B Aureli P Dorillo E Rosati E NOTCH and NF-κB interplay in chronic lymphocytic leukemia is independent of genetic lesion. Int J Hematol (2013) 98:153–7. 10.1007/s12185-013-1368-y 23690290

[20] Puente XS Beà S Valdés-Mas R Villamor N Gutiérrez-Abril J Martin-Subero JI Non-coding recurrent mutations in chronic lymphocytic leukaemia. Nature (2015) 526:519–24. 10.1038/nature14666 26200345

[21] Le Bras S Loyer N Le Borgne R. The multiple facets of ubiquitination in the regulation of notch signaling pathway. Traffic (2011) 12:149–61. 10.1111/j.1600-0854.2010.01126.x 21029288

[22] Baldoni S Del Papa B Dorillo E Aureli P De Falco F Rompietti C Bepridil exhibits anti-leukemic activity associated with NOTCH1 pathway inhibition in chronic lymphocytic leukemia. Int J Cancer (2018). 10.1002/ijc.31355 29508386 PMC6055653

