# Supplementary material for: NOTCH1 Is Aberrantly Activated in Chronic Lymphocytic Leukemia Hematopoietic Stem Cells
Source: Front Oncol. 2018 Apr 20;8:105. doi: 10.3389/fonc.2018.00105 (PMC5919960; doi:10.3389/fonc.2018.00105)
Supplement: Supplementary file 1 [file table_1.PDF]

## Supplemental Table 1. Characteristics of CLL patients

| Patient n.a | Age at diagnosis | Gender | Binet/Rai stage | IGVH status | Notch Mutation Allelic Burdern | Cytogenetic Alterations |
|---|---|---|---|---|---|---|
| CLL1 | 54 | F | IA | M | 44,52 | 17p del + Trisomy 12 |
| CLL2 | 53 | F | 0A | M | 18,5 | Normal |
| CLL3 | 71 | F | IIB | M | 25,12 | Normal |
| CLL4 | 66 | M | IIB | UM | 49 | 11q del |
| CLL5 | 78 | M | 0A | M | 41,18 | 13q del |
| CLL6 | 50 | F | 0A | UM | 25 | 13q del |
| CLL7 | 62 | M | IVC | M | 43 | Normal |
| CLL8 | 53 | M | 0A | UM | 6 | Normal |
| CLL9 | 70 | M | IIB | UM | 22 | 13q del |
| CLL10 | 75 | M | IVC | M | 12,25 | 11q del + Trisomy 12 |
| CLL11 | 50 | M | 0A | UM | 42 | Trisomy 12 |
| CLL12 | 50 | M | IIA | M | 49 | Normal |
| CLL13 | 65 | F | 0A | UM | 13,8 | 14q del + Trisomy 12 |
| CLL14 | 77 | M | 0A | UM | 16 | 13q del + Trisomy 12 |
| CLL15 | 64 | F | IIA | UM | 48 | 14q del |
| CLL16 | 71 | M | 0A | M | 0 | 13q del |
| CLL17 | 59 | M | IIB | M | 0 | Normal |
| CLL18 | 45 | M | IA | UM | 0 | 13q del |
| CLL19 | 68 | M | IA | UM | 0 | 11q del |
| CLL20 | 56 | M | IIB | M | 0 | 13q del |
| CLL 21 | 81 | M | IV C | M | 0 | 13q del |

Abreviations: M: male; F: female; UM: unmutated; M: mutated; del: deletion;
